# Relationship between general anesthesia and Alzheimer disease: A protocol for a systematic review and meta-analysis

**DOI:** 10.1097/MD.0000000000009314

**Published:** 2017-12-22

**Authors:** Geun Joo Choi, Hyun Kang, Chong Wha Baek, Yong Hun Jung, Jeong Wook Kim, Young Cheol Woo

**Affiliations:** a Department of Anesthesiology and Pain Medicine; b Department of Internal Medicine, Chung-Ang University College of Medicine, Seoul, Republic of Korea.

**Keywords:** Alzheimer disease, anesthesia, meta-analysis, protocol & guidelines, systematic review

## Abstract

**Background:**

Alzheimer disease (AD) entails a long-term progressive decline in the cognitive ability to think and remember, and it has become a major concern for patients receiving surgery and anesthesia. However, studies investigating the relationship between general anesthesia and AD have yielded inconsistent results. Therefore, we plan to perform a systematic review and meta-analysis to determine the relationship between general anesthesia and AD, and to verify whether general anesthesia is an independent risk factor for AD.

**Methods:**

A systematic and comprehensive search will be performed using MEDLINE, EMBASE, and Google scholar from their inception to August 2017. Peer-reviewed cohort and case-control studies including nested case-control studies reporting the relationship between general anesthesia and AD will be eligible for inclusion. The quality of included studies will be assessed using the Newcastle–Ottawa scale. Heterogeneity of estimates across studies as well as publication bias will be assessed. This systematic review and meta-analysis will be performed according to the Meta-analysis of Observational Studies in Epidemiology (MOOSE) guidelines and reported according to the Preferred Reporting Items for Systematic reviews and Meta-Analysis (PRISMA) guidelines. All statistical analyses will be conducted using the Stata SE version 15.0.

**Results:**

The results of this systematic review and meta-analysis will be published in a peer-reviewed journal.

**Conclusion:**

Our study will provide the evidence for the relationship between general anesthesia and dementia. The review will benefit patients and anesthesiologists, surgeons, and policymakers.

**Ethics and dissemination:**

Ethical approval and informed consent are not required, as the study will be a literature review and will not involve direct contact with patients or alterations to patient care.

**Trial registration:**

The protocol for this review has been registered in the PROSPERO network (registration number: CRD42017073790).

## Abstract 2

Supplemental Digital Content is available in the text

**Key Points**

- This systematic review and meta-analysis will provide a comprehensive and objective assessment of the relationship between general anesthesia and AD.
- The study will provide useful and novel information for patients, anesthesiologists, surgeons, and policymakers. The study will assess the methodological and reporting quality of included studies using the Newcastle–Ottawa scale, which can investigate the association of strength between general anesthesia and AD according to the quality of study.
- Subgroup analysis will be carried out based on study design, method of exposure measurement (self-reported vs medical records), risk of surgery (high-risk surgery vs mild- to moderate-risk surgery), and quality of study (low vs moderate to high), which enables us to investigate the association of strength according to the subgroups.
- One major limitation of our study protocol will be that many of the included studies may have poor methodological quality or may include insufficient explanation of their findings.

## 1 Introduction

As clinicians who perform anesthesia in various clinical fields, the authors of the present protocol have performed preanesthetic interviews with patients to evaluate medical history and patient condition, in order to appropriately plan for anesthesia and reduce patients’ perioperative anxiety and concerns related to surgery and anesthesia. During these interviews, one of the questions most frequently asked by patients is whether memory declines or risk for dementia increases following anesthesia and surgery. The present study was designed to answer these questions by critically reviewing current evidence and synthesizing the results.

Dementia defines a broad category of neurodegenerative diseases that damages neurons in areas essential to memory and reasoning, such as the hippocampus and the parietal and temporal lobes, thereby causing a long-term progressive decline in the cognitive ability to think and remember.^[1]^ It has been reported that dementia affected more than 35.6 million people worldwide in 2010,^[2]^ making it a substantial public health concern in modern society. The number of patients with dementia is expected to nearly double every 20 years, reaching 65.7 million in 2030 and 115.4 million in 2050.^[2]^

Of those types of dementia, Alzheimer's disease (AD) is the most common form of dementia, and account for 60 to 80 percent of dementia cases. It is estimated that 5.5 millions of Americans are living with AD in 2017.^[3]^

Although the precise causes, risk factors and pathogenesis of AD remain unclear, potential risk factors include advanced age, female gender, lower educational level, family history of AD, cardiovascular disease, depression, head trauma, and apolipoprotein E.^[4–7]^ In addition, the possibility has been raised that general anesthetics may induce neurotoxicity and lead to AD.

Fear of losing autonomy as well as the relatively high prevalence of AD in the older (age standardized prevalence for the older aged > 60 is 5–7% in the most world region) may be among the main reasons patients raise this concern during preanesthetic interviews.^[2]^

Although there have been reports that general anesthetics may not cause long-term neurocognitive outcome^[8]^ or may have dual effects (promotion vs protection) on dementia-associated neurotoxicity,^[9]^ animal and molecular studies provide credible evidence that exposure to general anesthetics may induce or exacerbate dementia.^[10,11]^ Preclinical studies have demonstrated that general anesthetics increase the accumulation of amyloid β protein^[12]^ and the hyper-phosphorylation of tau proteins,^[13]^ leading to neurofibrillary tangles in the brain, which suggests a possible mechanism whereby anesthesia may lead to dementia. However, human studies investigating the association between general anesthesia and dementia have yielded inconsistent results.

A reanalysis of 8 case-control studies performed by EURODEM Risk Factors Research Group^[14]^ and a meta-analysis of 15 case-control studies^[15]^ did not show an association between general anesthesia and dementia. However, considerable variation was observed between studies in terms of data collection methodology. In addition, these meta-analyses incorporated nonpeer reviewed articles and included only case-control studies, which are prone to limitations such as recall bias.^[16]^

Several large-scale cohort studies^[17,18]^ and high-quality case-control studies^[19,20]^ have been published since the most recent meta-analyses were conducted, and some of these new publications report a greater association between general anesthesia and dementia compared to earlier studies.^[17,20]^ An updated meta-analysis is, therefore, needed to synthesize and critically review the current evidence on this research question.

The objectives of this systematic review and meta-analysis are to determine the association between the administration of general anesthesia and AD, and to verify whether anesthesia is an independent risk factor for dementia.

## 2 Methods and analysis

Our systematic review and meta-analysis protocol were developed following the preferred reporting items for systematic review and meta-analysis protocols (PRISMA-P) statement.^[21]^ The protocol for this review has been registered in the PROSPERO network (registration number: CRD42017073790). This systematic review and meta-analysis of the association between general anesthesia and AD will be performed according to the Meta-analysis of Observational Studies in Epidemiology (MOOSE) guidelines^[22]^ and will be reported according to the Preferred Reporting Items for Systematic reviews and Meta-Analysis (PRISMA) guidelines.^[23]^

### 2.1 Inclusion and exclusion criteria

#### 2.1.1 Types of studies

Peer-reviewed cohort and case-control studies including nested case-control studies will be eligible for inclusion. No language or date restrictions will be applied. Review articles, case reports, case series, letters to the editor, commentaries, proceedings, laboratory science studies, and any other nonrelevant studies will be excluded from analysis.

#### 2.1.2 Population

Inclusion criteria for study populations will be as follows: (1) the older (defined as more than 60 or 65 years) from all countries and (2) who has not been diagnosed with AD before the beginning of the study period. If the study defined the older other than 60 or 65 years of age, an attempt will be made to contact the study authors to obtain the relevant information. When unsuccessful, pooled analysis will be performed including the data of that study first, and then sensitivity analysis will be performed excluding the data. No restrictions will be applied in terms of sex, race/ethnicity, or socioeconomic status.

#### 2.1.3 Intervention

Interventions to be examined will include any exposure to general anesthesia for surgery. General anesthesia will include anesthesia performed using only inhalational anesthetics. Intravenous anesthesia, spinal anesthesia, epidural anesthesia, regional anesthesia will be excluded. If an article reports on general anesthesia including intravenous, spinal, epidural or regional anesthesia along with inhalation anesthesia, we will try to contact the study authors to obtain information on general anesthesia using inhalation anesthetics. When unsuccessful, data will be analyzed on general anesthesia including intravenous, spinal, epidural or regional anesthesia, and then sensitivity analysis will be performed excluding the data. The information for the data source for exposure (self-reported vs medical record) will be collected.

#### 2.1.4 Comparison

Comparison groups will include individuals with no history of any type of anesthesia. If a study investigates the associations of AD and general anesthesia using 2 or more comparison groups (e.g., hospital control group and community control group), and reports each outcome separately, pooled estimates of associations for these groups will be calculated and used for analysis. If the study reports outcomes for only 1 comparison group out of 2 or more examined, we will try to contact the study author to obtain the information not reported. When unsuccessful, the reported value will be used for our analysis.

#### 2.1.5 Outcomes measures

We will include dementia case diagnosed using standard criteria (such as International List of Causes of Death, Diagnostic and Statistical Manual of Mental Disorders (DSM) or National Institutes of Neurological and Communicative Disorders and Stroke—Alzheimer's Disease and Related Disorders (NINDS-ADRDA)) or clinically diagnosed by medical staff.

Studies reporting odds ratios (OR), relative risks (RR), or hazard ratios (HR) for dementia (unadjusted or adjusted), or providing the number of individuals with and without dementia (from which ORs can be calculated) will be included.

### 2.2 Information sources

#### 2.2.1 Electronic search

A search will be performed in MEDLINE, EMBASE, and Google scholar using search terms related to general anesthesia and dementia. Search terms to be used for MEDLINE and EMBASE are presented in the Appendix. Two authors will screen titles and abstracts of the retrieved articles. Reference lists will be imported into Endnote software (Thompson Reuters, CA) and duplicate articles removed. Additional relevant articles will be identified by scanning the reference lists of articles found from the original search.

#### 2.2.2 Study selection

The titles and abstracts identified through the search strategy described above will be scanned independently by 2 authors. To minimize data duplication as a result of multiple reporting, papers from the same author will be compared. For reports determined to be eligible based on the title or abstract, the full paper will be retrieved. Potentially relevant studies chosen by at least 1 author will be retrieved and evaluated in full-text versions. Articles meeting the inclusion criteria will be assessed separately by 2 authors, and any discrepancies will be resolved through discussion. In cases where agreement cannot be reached, the dispute will be resolved with the help of a third investigator. A flow diagram for the search and selection process will be developed following PRISMA guidelines.

### 2.3 Data extraction

Using a standardized extraction form, the following data will be extracted independently by 2 authors: study name (along with the name of the first author and year of publication), country where the study was conducted, source from which patients or study participants were selected, study design, exposure definition, method of exposure measurement (self-reported vs medical records), outcome definition, risk of surgery, definition of the older, RR, OR, or HR with 95% confidence intervals (CIs), methods for controlling covariates and confounding variables controlled for, number of cases/controls or cohort groups, and total number of participants.

If information is missing, an attempt will be made to contact the study authors to obtain the relevant information. When unsuccessful, missing information will be calculated if possible from the relevant data within the study.

The reference list will be divided in half, and 2 authors will complete data extraction for each half of the list. Data extraction forms will then be cross-checked to verify accuracy and consistency of extracted data.

### 2.4 Study quality assessment

The quality of the studies will be independently assessed by 2 authors using the Newcastle–Ottawa scale (NOS), a validated quality assessment instrument for nonrandomized trials which assesses 3 parameters of study quality: selection, comparability, and exposure assessment.^[24]^ Any discrepancies will be resolved through discussion. If agreement cannot be reached, the dispute will be resolved with the help of a third investigator. The NOS assigns a maximum score of 4 for selection, 2 for comparability, and 3 for exposure, for a maximum total score of 9. Studies with a total NOS score of 5 or greater are considered to be of moderate to high quality, whereas those with an NOS score of less than 5 are considered low-quality studies.

### 2.5 Statistical analysis

Ad-hoc tables will be designed to summarize data from the included studies and show their key characteristics and any important questions related to the aim of this review. After data have been extracted, reviewers will determine whether a meta-analysis is possible.

#### 2.5.1 Data synthesis

The pooled OR for all studies with corresponding 95% CI will be computed. If cohort studies report only HR with corresponding 95% CI, the pooled HR will be computed for cohort studies and the pooled OR will be computed for case-control studies.

Between-study heterogeneity will be assessed using the Cochran's Q and Higgins's *I*^2^ statistics. A *P*-value of < .10 for the chi^2^ statistic or an *I*^2^ greater than 50% will be considered as showing considerable heterogeneity, and data will be analyzed using the Mantel–Haenszel random-effect model. Otherwise, we will apply the Mantel–Haenszel fixed-effect model.^[25]^

#### 2.5.2 Sub-group analysis

Sub-group analysis will be carried out based on study design, method of exposure measurement (self-reported vs medical records), risk of surgery (high-risk surgery vs mild- to moderate-risk surgery), and quality of study (low vs moderate to high). If cohort studies report only HRs, subgroup analysis based on study design will not be performed.

#### 2.5.3 Sensitivity analysis

We will conduct sensitivity analyses to evaluate the influence of individual studies on the overall effect estimate by excluding 1 study at a time from the analysis. We will also conduct the sensitivity analysis excluding the study which use the criteria for the older other than 60 or 65 years of age or which reports on general anesthesia including intravenous, spinal, epidural, or regional anesthesia along with inhalation anesthesia

#### 2.5.4 Publication bias

Publication bias will be assessed by using Begg's funnel plot and Egger's test. Begg's funnel plots are scatter plots of the log ORs of individual studies on the *x*-axis against 1/standard error (SE) of each study on the *y*-axis. Egger's test is a test for linear regression of the normalized effect estimate (log OR/SE) against its precision (1/SE).^[26]^ An asymmetrical funnel plot or a *P*-value of < .1 from Egger's test will be considered to indicate the presence of publication bias. If publication bias is detected, trim and fill analyses will be performed. All statistical analyses will be performed using Stata SE version 15.0 (StataCorp, College Station, TX).

### 2.6 Evidence synthesis

The evidence grade will be determined using the guidelines of the GRADE (Grading of Recommendations, Assessment, Development, and Evaluation) system which uses sequential assessment of the evidence quality that is followed by an assessment of the risk–benefit balance and a subsequent judgment on the strength of the recommendations.^[27]^

## 3 Discussion

The objectives of this systematic review and meta-analysis are to determine the relationship between general anesthesia and dementia and to verify whether anesthesia is an independent risk factor for dementia.

As techniques for surgery and anesthesia have advanced, both the duration and quality of life have been improved. Thus, the number of surgeries performed under anesthesia has increased, resulting in increased concerns regarding side effects from surgery and anesthesia. Among these side effects, the possibility of an association between general anesthesia and dementia is now an ongoing concern.

Two meta-analyses on this research question have been previously published,^[14,15]^ and several additional studies have been published since. The 2 published meta-analyses included only case-control studies, which are prone to biases, in addition to nonpeer reviewed articles. In addition, there was considerable variation between studies in terms of data collection methodology.

To respond to patients’ concerns appropriately and precisely, careful consideration of the research to date and future evidence-based recommendations will be needed. We, therefore, planned this systematic review and meta-analysis to summarize and assess the published evidence to date.

We anticipate some limitations to our analysis. First, randomized controlled trials cannot be performed for ethical reasons. Thus, we will include only observational studies. Second, definitions for exposure and outcome and data sources are expected to vary between studies, and the resulting heterogeneity will to some extent limit the conclusions that can be drawn from the meta-analysis. Third, surgery itself may also contribute to the increase in AD risk. High-risk surgeries such as cardiac surgery may raise the risk for delirium and postoperative cognitive dysfunction.^[28,29]^ Consequently, there is a potential for increased risk for dementia in high risk surgery. However, as surgeries are nearly always performed under anesthesia, it is very difficult to separate surgery and anesthesia, thus to analyze the risk of surgery and anesthesia separately. We will try to explore if the risk of surgery will contribute the risk of AD by comparing high-risk group and moderate or mild-risk group. Fourth, surgery related risk factors other than general anesthesia such as comorbidity may limit to draw meaningful conclusion. These limitations will be addressed in the discussion section of the systematic review and will be taken into consideration when drawing conclusions from our study.

### 3.1 Ethics and dissemination

#### 3.1.1 Ethical issues

This systematic review does not require ethics approval or obtaining informed consent because there will be no direct contact with individual patients, and only previously published data will be included in the review.

#### 3.1.2 Publication plan

This systematic review will be published in a peer-reviewed journal and will be disseminated electronically and in print.

## Supplementary Material

Supplemental Digital Content
