# Supplementary material for: Relationship between general anesthesia and Alzheimer disease: A protocol for a systematic review and meta-analysis
Source: Medicine (Baltimore). 2017 Dec 22;96(51):e9314. doi: 10.1097/MD.0000000000009314 (PMC5758206; doi:10.1097/MD.0000000000009314)
Supplement: Supplemental Digital Content [file medi-96-e9314-s001.docx]

**Appendix: Search terms to be used**

**Medline**

1. Exp dementia/
2. Dementia.mp.
3. Amentia.mp.
4. Alzheimer.mp.
5. or/1-4
6. expanaesthesia/
7. exp anesthetics/
8. an?esthesia.mp.
9. anesth*.mp.
10. anaesth*.mp.
11. Or/6-10
12. 5 and 11
13. Case report.tw.
14. Letter.pt.
15. Historical article.pt.
16. Review.pt.
17. or/13-16
18. 12 not 17

**EMBASE**

1. 'dementia'/exp
2. Dementia
3. Amentia
4. 'alzheimer disease'
5. #1 OR #2 OR #3 OR #4
6. 'anaesthesia'/exp
7. 'anesthetic agent'/exp
8. anaesthesia
9. anaesthetic.mp.
10. #6 OR #7 OR #8 OR #9
11. #5 AND #10
12. 'case study'/exp
13. 'case report'/exp
14. 'abstract report'/exp
15. 'letter'/exp
16. #12 OR #13 OR #14 OR #15
17. #11 NOT #16
